# RNA sequencing reveals CircRNA expression profiles in chicken embryo fibroblasts infected with velogenic Newcastle disease virus

**DOI:** 10.3389/fvets.2023.1167444

**Published:** 2023-03-29

**Authors:** Libin Chen, Jiayu Ruan, Yiyi Chen, Wenxuan Deng, Jinyu Lai, Lei Fan, Juncheng Cai, Chan Ding, Qiuyan Lin, Bin Xiang, Tao Ren

**Affiliations:** ^1^College of Veterinary Medicine, South China Agricultural University, Guangzhou, China; ^2^Key Laboratory of Animal Vaccine Development, Ministry of Agriculture, Guangzhou, China; ^3^National and Regional Joint Engineering Laboratory for Medicament of Zoonosis Prevention and Control, Guangzhou, China; ^4^Key Laboratory of Zoonosis Prevention and Control of Guangdong Province, Guangzhou, China; ^5^Shanghai Veterinary Research Institute (SHVRI), Chinese Academy of Agricultural Sciences (CAAS), Shanghai, China; ^6^College of Veterinary Medicine, Yunnan Agricultural University, Kunming, Yunnan, China

**Keywords:** chicken embryo fibroblasts, Newcastle disease virus, RNA sequencing, infection, circular RNA

## Abstract

**Introduction:**

Newcastle disease virus (NDV) is an important avian pathogen prevalent worldwide; it has an extensive host range and seriously harms the poultry industry. Velogenic NDV strains exhibit high pathogenicity and mortality in chickens. Circular RNAs (circRNAs) are among the most abundant and conserved eukaryotic transcripts. They are part of the innate immunity and antiviral response. However, the relationship between circRNAs and NDV infection is unclear.

**Methods:**

In this study, we used circRNA transcriptome sequencing to analyze the differences in circRNA expression profiles post velogenic NDV infection in chicken embryo fibroblasts (CEFs). Gene ontology (GO) and Kyoto Encyclopedia of Genes and Genomes (KEGG) enrichment analyses were used to reveal significant enrichment of differentially expressed (DE) circRNAs. The circRNA- miRNA-mRNA interaction networks were further predicted. Moreover, circ-EZH2 was selected to determine its effect on NDV infection in CEFs.

**Results:**

NDV infection altered circRNA expression profiles in CEFs, and 86 significantly DE circRNAs were identified. GO and KEGG enrichment analyses revealed significant enrichment of DE circRNAs for metabolism-related pathways, such as lysine degradation, glutaminergic synapse, and alanine, aspartic-acid, and glutamic-acid metabolism. The circRNA- miRNA-mRNA interaction networks further demonstrated that CEFs might combat NDV infection by regulating metabolism through circRNA-targeted mRNAs and miRNAs. Furthermore, we verified that circ-EZH2 overexpression and knockdown inhibited and promoted NDV replication, respectively, indicating that circRNAs are involved in NDV replication.

**Conclusions:**

These results demonstrate that CEFs exert antiviral responses by forming circRNAs, offering new insights into the mechanisms underlying NDV-host interactions.

## 1. Introduction

Newcastle disease virus (NDV) belongs to the Paramyxoviridae family and is a highly contagious avian pathogen, causing febrile illness in various birds; chickens are especially infected (1). Genotype VII velogenic NDVs are the most harmful NDV strains affecting the poultry industry in China in the past decade, causing massive die-offs of chicken flocks (2, 3). Although the number of genotype VII NDV infections has decreased owing to mass vaccination, it still poses a threat. In recent years, research on the pathogenesis of NDV has progressed, but there are still unanswered questions, limiting the prevention and control of the virus.

Circular RNAs (circRNAs) are covalently closed non-coding RNAs in eukaryotes, which are mainly derived from reverse splicing during mRNA synthesis (4). CircRNAs possess a covalently closed loop without 5′ or 3′ polarities and a poly(A) tail, conferring greater resistance against to exonucleases (5). Around 2010, with advances in high-throughput RNA sequencing (RNA-seq) technology, eukaryotes (e.g., plants, animals, and humans) were discovered to produce thousands of circRNAs (6, 7). CircRNAs regulate transcriptional silencing, translation, and degradation of specific mRNAs by acting as miRNA “sponges” (8, 9). Moreover, circRNAs can act as protein scaffolds and recruit other RNA species (10). In the past decade, circRNAs have been found to play an important role in host–virus interactions, specifically in the antiviral immune response (11). CircRNAs were found to be involved in regulating the replication of viruses, such as the Epstein-Barr virus (12), influenza virus (13) and Hantaan virus (14). However, the specific roles of circRNAs in the pathogenesis of velogenic NDV remain largely unknown.

In this study, RNA-seq was used to determine and analyze circRNA expression profiles post-NDV infection in chicken embryo fibroblasts (CEFs). Furthermore, the functions of enriched circRNAs were explored. The study results suggested that circRNAs regulate NDV replication in chickens, providing a theoretical basis for further research on the pathogenesis of NDV.

## 2. Materials and methods

### 2.1. Viruses and cells

The genotype VII velogenic NDV strain GM (chicken/Guangdong/GM/2014; GenBank ID: DQ486859.1) was preserved in our laboratory (15), multiplied in 9-day-old embryonated specific-pathogen-free (SPF) chicken eggs, and stored at −80°C. Determination of TCID_50_ was conducted as described previously (16). CEFs were prepared from 10-day-old embryonated eggs as previously described (17).

### 2.2. Construction of circRNA library

At 24 h post-infection with 1 multiplicity of infection (MOI) GM strain, total RNA was extracted from infected and control CEFs cells using TRIzol reagent. Agarose gel electrophoresis can be used in testing the integrity of the samples. Meanwhile, the A260/A280 ratio for the Nanodrop detection of RNA should be in the range from 1.8 to 2.0. Following rRNA depletion with Illiumina Ribo Zero Gold Kit and liner RNA degradation with RNase R, circRNAs were broken into short sections. A complete cDNA was obtained by the addition of random hexamers, buffer, dNTPs, RNase H and DNA polymerase. To establish a whole library, the system was first configured according to the QiaQuick PCR kit protocol, and thereafter, the fragment matching the length of the target gene was recovered. Finally, validation by Illumina HiSeqTM 2500 sequencing indicated that the rRNA library has been successfully constructed.

### 2.3. Analysis of circRNA quantification and differential expression

The raw reads obtained by high-throughput sequencing were screened. Removed reads containing adapters, finished preliminary filtering of low-quality reads containing more than 50% of useless bases and reads containing more than 10% of unknown nucleotides. The remaining unfiltered readings are prepared for circRNA identification. Next, 20 m from both ends of the unmapped reads were extracted and aligned with the reference genome to detect unique anchor positions within splice site. Anchor reads alighting in the reversed orientation (head-to tail) indicated circRNA splicing and were subjected to find_circ to identify circRNAs. Reads identified as circRNAs were further expanded so that the circRNA sequence inside the breakpoint of the read could be complete and surrounded by GU/AG splice sites on both sides of the breakpoint. For quantifying circRNAs, back-spliced junction reads were scaled to reads per million (RPM) mapped reads, using the following formula:


$$\begin{array}{l} RPM=\frac{10^{6}C}{N} \end{array}$$

In this formula, C is the number of back-spliced junction reads that are. uniquely aligned to a circRNA, and N is the total number of back-spliced junction reads. Therefore, the calculated expression can be directly used for comparing the differential expression among samples.

### 2.4. Gene ontology (GO) and Kyoto encyclopedia of genes and genomes (KEGG) pathway enrichment analysis

GO enrichment analyses provided all GO terms for which source genes were significantly enriched compared to the genomic background and filtered source genes corresponding to biological functions. KEGG pathway enrichment analysis identified significantly enriched metabolic pathways or signal transduction pathways in source genes compared with the whole genome background. To analyze the functions of the differentially expressed circRNAs (DE circRNAs), we performed GO and KEGG pathway enrichment analyses on the parental genes of the DE circRNAs using the Database for Annotation Visualization and Integrated Discovery (https://david.ncifcrf.gov/) (18) and the KEGG database (http://www.genome.jp/kegg/) (19).

### 2.5. miRNA sponge analysis and integrated analysis of circRNAs-miRNAs-mRNAs

For circRNAs that have been annotated in circBase, the target relationship with miRNAs can be predicted by StarBase (v2.0). For novel circRNAs, target genes of samples were predicted by using Mireap, Miranda (v3.3a) and TargetScan (v7.0). To predict mRNAs interacting with circRNAs and miRNAs, miRTarBase (v6.1) was used to predict mRNAs targeted by the miRNAs sponge. The resulting correlation of circRNAs-miRNAs-mRNAs and the resulting circRNAs-miRNAs-mRNA relationship network were visualized using Cytoscape.

### 2.6. Western blot and RT-qPCR

CEFs were infected with 1 MOI GM strain and harvested at indicated time points. The expression of NP protein was detected using western blot, as described previously (20).

Using 1,000 ng RNA as a template, Reverse Transcriptase M-MLV reverse transcriptase (TaKaRa, Dalian, China) and other reagents were added in a 1.5-mL tube. Samples were kept in a 42°C constant-temperature water bath for 10 min and inactivated for 2 min in 95°C, cDNA was obtained and stored at −20°C. Primers were designed according to the serial number of the GenBank chicken source gene (Table 1). Divergent primers were designed based on the circRNA predicted sequences provided by the transcriptome. Amplification was performed with ChamQ Universal SYBR qPCR Master Mix (Vazyme, Nanjing, China) by RT-PCR. After the reaction, the transcription level was calculated by formula 2^−(Δ*Δct*)^; the values were compiled into a histogram using GraphPad Prism 8 software (GraphPad Software, Inc., USA), and a difference analysis was performed.

**Table 1:** Primers used in this study.

**Primer**	**Sequence (5^′^ → 3^′^)**	**Length of product**
con-003546-F	GAGGCATCTCCGTATTC	427 bp
con-003546-R	TAGGTCCCGTGTTAAAC	
div-003546-F	GACACAGTTCTGGACCTCGC	314 bp
div-003546-R	TCACTCATGGCTGTGGACTC	
con-002830-F	TGGAACCCAGACCTTTGATGA	379 bp
con-002830-R	GTGGCCACCCTTTTCATACG	
div-002830-F	CAAGACAGACACAGGGCAAGA	194 bp
div-002830-R	ATCTGCCTCCACTGGTAAGAG	
con-005418-F	GACTGAGGCAGCTCAAGAGA	465 bp
con-005418-R	GACCAAGTGCATTGACCAGC	
div-005418-F	ACACTTTTCTGTAGGCGCTG	286 bp
div-005418-R	CGCAAAGAGCTCACAGAAGTC	
con-000636-F	GATTTGTGACTTCGGACTGG	232 bp
con-000636-R	AAGGATATGGTTAAGCTGGT	
div-000636-F	GGATCCCCTTCCCAAGAAGATT	212 bp
div-000636-R	CAGAGTTCTCTGGCAGTACG	
con-001830-F	TATGCCTGCGACTGGATAGC	149 bp
con-001830-R	TGTAGCGCCAAGAGGAGAAC	
div-001830-F	CCCATGGTGGCAGGTTCTC	238 bp
div-001830-R	CTTCGACAGATCCACTGGCTT	

### 2.7. Sanger sequencing (circRNA)

Reverse spliced region of circRNA was amplified by using divergent primers. The products recovered were sent to Sangon Biotech for Sanger sequencing, and the sequencing results were aligned by NCBI Blast analysis, MegAlign and SnapGene.

### 2.8. Nucleoplasm isolation and RNA extraction

Samples were subjected to nucleocytoplasmic separation according to the instructions. RNA was extracted from cells using Trizol reagent.

### 2.9. siRNA interference test

For transfection, cells cultured in 6-well plates were washed three times with PBS and transfected with siRNA (Table 2) using Lipofectamine TM 2000 (Invitrogen) according to the manufacturer's protocol.

**Table 2:** SiRNAs for transfection.

**siRNA**	**Sequences (5^′^ → 3^′^)**
si-circEZH2-1	TGCTTCTTACATCGTTAGT
si-circEZH2-2	CTTCTTACATCGTTAGTCA
si-circEZH2-3	CATCGTTAGTCATGGGTCA

### 2.10. Statistical analysis

Data presentation and statistical analyses were performed using GraphPad Prism 8 software (GraphPad Software, Inc., USA). Data were expressed as mean ± standard deviation (SD). Data were analyzed using Student's *t*-test for pairwise comparisons or an analysis of variance/Dunn multiple comparison test for multiple comparisons. Statistical significance was set at *p* < 0.05, *p* < 0.01, and *p* < 0.001 for values that were significant, very significant, and highly significant, respectively.

## 3. Results

### 3.1. Characterization of the circRNA expression profile post-NDV infection in CEFs

**Figure 1:**
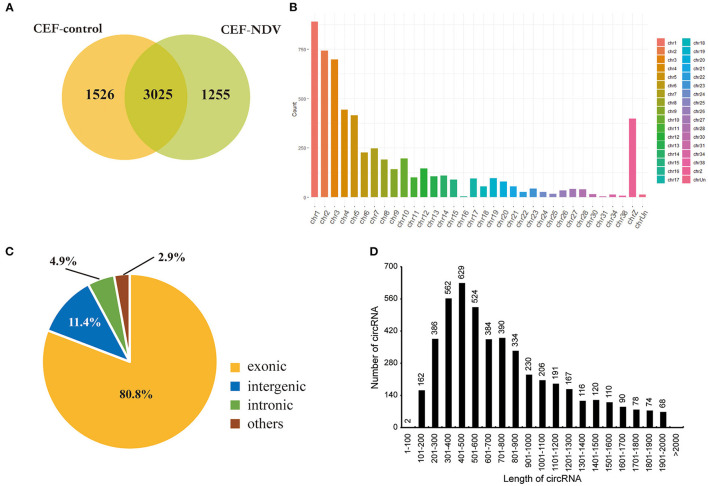
CircRNA expression profile in CEFs. **(A)** circRNAs expressed in CEFs with or without NDV infection. Using find_circ performs circular RNA identification on sequencing results. **(B)** circRNAs expressed on each chromosome in CEFs. **(C)** Different circRNAs expressed in CEFs. **(D)** Length of circRNAs expressed in CEFs.

We infected CEFs with NDV at 1 MOI, and lesions appeared 9 h post-infection. This timepoint was therefore selected to analyze circRNA transcriptomes. Mock-inoculated CEFs 9 h post-infection were used as the negative control. For the circRNA transcriptome sequencing, three replicate cell samples were selected from the negative control and experimental groups. The samples were subjected to next-generation sequencing, and after data purification, 5,806 circRNAs were identified in the CEFs. The number of circRNA intersections between the control and infected groups was 3,025 (Figure 1A). The length, chromosomal distribution, and type of circRNAs were similar between the control and experimental groups. Most circRNAs were distributed on chromosome 1, followed by chromosomes 2 and 3; >50% of circRNAs were distributed on chromosomes 1 to 5 (Figure 1B). CircRNAs of CEFs were mainly located in exons (Figure 1C). The majority of circRNAs were 401–500 bp long, and the smallest fraction was 1–200 bp long. CircRNAs did not exceed >2,000 bp in length (Figure 1D). Overall, we successfully identified circRNAs in CEFs, and velogenic NDV infection was found to affect circRNA expression in CEFs.

### 3.2. Identification and verification of significantly DE circRNAs

To investigate the antiviral response to NDVs in CEFs, we analyzed the circRNA expression profile and screened significantly DE circRNAs (fold change >2 and *p* < 0.05). A total of 86 (44 upregulated and 42 downregulated) DE circRNAs were identified in the CEF-control group vs. experimental group (Supplementary Table 1).

**Figure 2:**
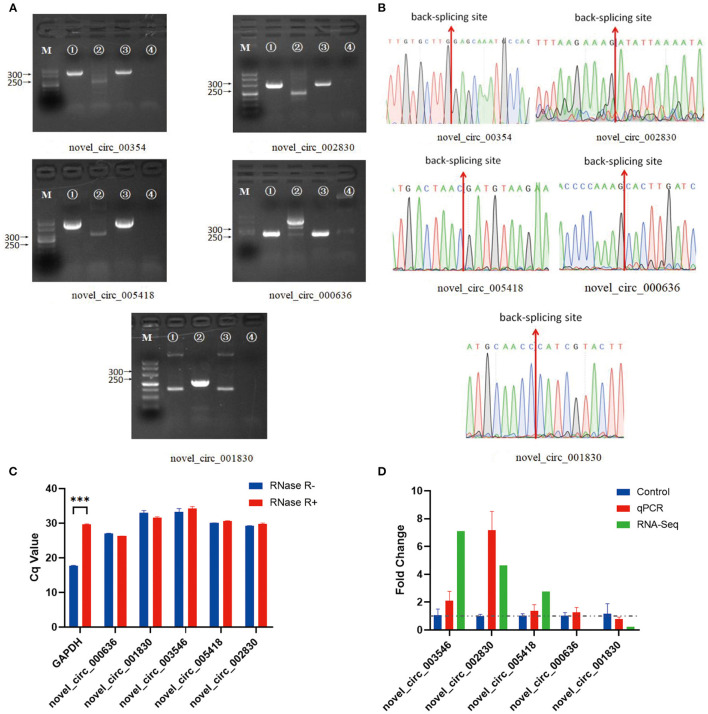
Verification of five significantly DE circRNAs in the NDV-infected DEF cell group. **(A)** Electrophoretogram of the PCR products. M: DNA marker 500; 1: Convergent primers were used for amplification using cDNA as a template; 2: Divergent primers were used for amplification using cDNA as a template; 3: Convergent primers were used for amplification using gDNA as a template; 4: Divergent primers were used for amplification using gDNA as a template. **(B)** The head-to-tail junctions of significantly DE circRNAs were confirmed by Sanger sequencing. The red line indicates the back-splicing site position. The area from left (or right) of the site is the end (or start) sequence of the circRNAs (junction). **(C)** qRT-PCR was used to detect significantly DE circRNAs resistant to RNase R digestion. Glyceraldehyde-3-phosphate dehydrogenase (*GAPDH*) was used as a linearity control. Data are expressed as mean ± SEM (*n* = 3). **(D)** Validation of significantly DE circRNAs in the NDV-infected DEF cell group using qRT-PCR. Data are expressed as mean ± SEM (*n* = 3).

In the experimental group, we validated five significantly DE circRNAs, including three upregulated (novel_circ_003546, novel_circ_002830, and novel_circ_005418) and two downregulated (novel_circ_000636 and novel_circ_001830) circRNAs (Table 3), using reverse transcription PCR (RT-PCR), Sanger sequencing, and RNase R digestion. We designed convergent and divergent primers for validating circRNAs; the specific sites and size of the PCR products differed. RT-PCR did not amplify the fragment of interest using divergent primers with gDNA as a template (Figure 2A, lane 4), whereas the other lines all amplified the band of interest (Figure 2A, lanes 1–3). This result suggested that circRNAs were circular and derived from post-transcriptional mRNA precursors by alternative splicing. Sanger sequencing of the amplified products with divergent primers using cDNA as a template showed that the sequencing results were consistent with the back-splicing region alignment (Figure 2B). Furthermore, qRT-PCR validation using RNase R-treated RNA showed that the Cq value of GAPDH was significantly higher in the experimental group than in the control group. This finding indicated that the amount of housekeeping mRNAs was significantly reduced after RNase R treatment, whereas the expression of selected circRNAs did not change significantly after RNase R digestion (Figure 2C). These five circRNAs were all RNase R-enzyme tolerant with stable circular structures. qRT-PCR results showed that the upregulation of three and downregulation of two circRNAs were consistent with the transcriptome sequencing results in the CEFs of the experimental group (Figure 2D). These results confirm that the circRNA identification in this study was accurate and reliable.

**Table 3:** CircRNAs selected for expression verification.

**CircRNA**	**Host source gene**	**Length**	**Type**	***p*-value**	**|log2FC|**	**Up/Downregulation**
novel_circ_003546	LPAR1	916	annot_exons	0.02	2.83	Up
novel_circ_002830	RNF13	710	annot_exons	0.00	2.21	Up
novel_circ_005418	EZH2	899	annot_exons	0.03	1.47	Up
novel_circ_000636	ERK	737	annot_exons	0.04	17.89	Down
novel_circ_001830	BANP	512	annot_exons	0.04	2.05	Down

### 3.3. KO and KEGG enrichment analyses of significantly DE circRNA

**Figure 3:**
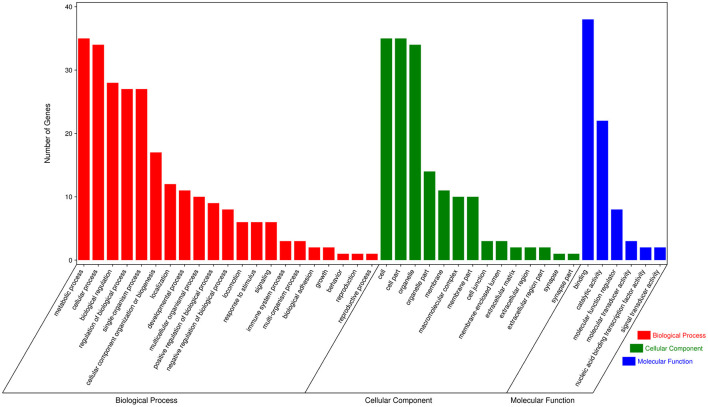
GO annotation of significantly DE circRNAs from host source genes. GO annotation of significantly DE circRNAs from NDV-infected CEFs.

**Figure 4:**
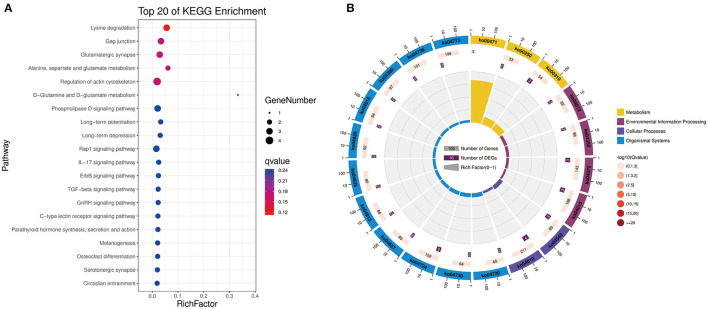
KEGG pathway annotation of significantly DE circRNAs from host source genes. **(A)** Gradient map for KEGG pathway annotation of significantly DE circRNAs from NDV-infected CEFs. **(B)** Circular map for KEGG pathway annotation of significantly DE circRNAs from NDV-infected CEFs.

To further explore the functions of significantly DE circRNAs during NDV infection in CEFs, the host source genes of these circRNAs were used for GO and KEGG enrichment analyses. For GO-biological processes (BP), GO-cellular component (CC), and GO-molecular function (MF), the significantly DE circRNAs were mainly associated with metabolic, cellular, and biological regulation processes; cell, cell part, and organelle; and binding and catalytic activities, respectively (Figure 3). GO enrichment analysis indicated that the circRNAs in CEFs respond to NDV infection by regulating different gene functions. KEGG enrichment analysis showed that the significantly DE circRNAs in the experimental group were mainly enriched in lysine degradation, glutaminergic synapse, the metabolism of alanine, aspartic acid, and glutamic acid, and regulation of actin cytoskeleton (Figure 4A). These results indicated that circRNAs were mainly involved in metabolism-related regulatory pathways in the experimental group, and circRNAs may respond to viral infection by regulating cell metabolism. Additionally, circRNAs were mainly enriched in organismal systems (Figure 4B).

### 3.4. Establishment of circRNA-miRNA-mRNA interaction networks

**Figure 5:**
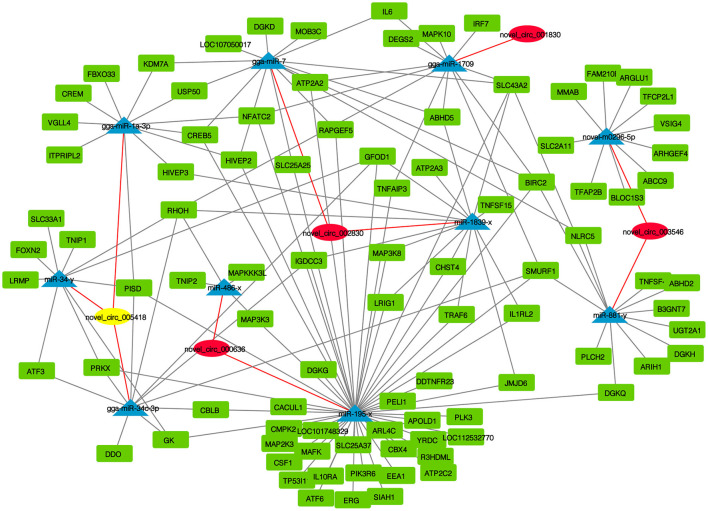
CircRNA-miRNA-mRNA interaction network of significantly DE circRNAs from NDV-infected CEFs.

CircRNAs function as miRNA sponges and inhibit target gene degradation by miRNAs mediating different biological processes during virus-induced cancer and infection. In total, 102 bound miRNAs were predicted in significantly DE circRNAs in the experimental group. The miRNA genes binding mRNAs were predicted to construct circRNA-miRNA-mRNA interaction networks; circRNAs of CEF targeted multiple genes related to metabolism, such as GK, CMPK2, PISD, CBLB, DGKQ, RHOH, and DDO (Figure 5). These results suggest that CEFs might combat NDV infection by regulating metabolism through circRNA-targeted mRNAs and miRNAs. Among them, we found that the above-mentioned mRNAs were mainly targeted by gga-miR-1a-3p, gga-miR-34c-3p, and miR-34-y, while novel_circ_005418 (circ-EZH2) targeted those miRNAs, indicating that circ-EZH2 might have potential to regulate relevant antiviral immune processes.

### 3.5. CircRNA-EZH2 affected NDV infection in CEFs

**Figure 6:**
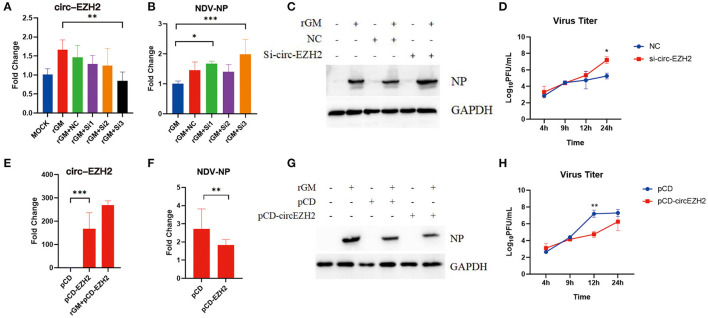
NDV viral proliferation after circ-EZH2 knockdown or overexpression. **(A)** qRT-PCR analysis to verify siRNA efficiency of the circ-EZH2 knockdown. **(B)** qRT-PCR analysis to measure NP gene production post-NDV infection during circ-EZH2 knockdown. **(C)** Western blot analysis to measure NP gene production post-NDV infection during circ-EZH2 knockdown. **(D)** NDV replication post NDV infection during circ-EZH2 knockdown. **(E)** qRT-PCR analysis to verify circ-EZH2 overexpression. **(F)** qRT-PCR analysis to measure NP gene production post-NDV infection during circ-EZH2 overexpression. **(G)** Western blot analysis to measure NP gene production post-NDV infection during circ-EZH2 overexpression. **(H)** NDV replication post NDV infection during circ-EZH2 expression.

The previous enrichment and interaction network analysis results showed that circ-EZH2 regulated multiple pathways and functional genes during NDV infection. Therefore, circ-EZH2 was selected to determine its effect on NDV infection in CEFs. Plasmids and siRNAs targeting circ-EZH2 were constructed, and the impact of NDV proliferation after circ-EZH2 knockdown or overexpression was investigated (Figures 6A, E). qRT-PCR (Figure 6B) and western blot (Figure 6C) results indicated that circ-EZH2 knockdown promoted the gene and protein expression of NDV nucleocapsid protein (NP), while its overexpression decreased it (Figures 6F, G). NDV viral growth curves showed similar results, with significantly higher and lower viral titers at 12–24 h post-infection after circ-EZH2 knockdown and overexpression, respectively (Figures 6D, H). These results were consistent with the transcriptome sequencing results, suggesting that circ-EZH2s play a role in NDV inhibition during infection in CEFs.

### 3.6. KEGG enrichment analyses of circ-EZH2-targeted mRNAs

Our study showed that circ-EZH2 promoted resistance to NDV infection in CEFs. We performed enrichment analysis of mRNAs targeted by the miRNAs predicted to bind circ-EZH2. Circ-EZH2 was predicted to bind gga-miR-1a-3p, gga-miR-34c-3p, and miR-34-y, targeting 84 mRNAs. These 84 target genes were subjected to KEGG enrichment analysis, which showed that targeted mRNAs were mainly enriched in the C-type lectin receptor signaling pathway and adrenergic signaling in cardiomyocytes (Table 4). It is worth mentioning that the C-type lectin receptor signaling pathway is closely related to the antiviral immune response (21).

**Table 4:** KEGG enrichment analyses of mRNA targeted by miRNA binding with circ-EZH2.

**Enriched pathways**	**Enriched genes**	***p*-value**
C-type lectin receptor signaling pathway	3	5.10 × 10^−2^
Adrenergic signaling in cardiomyocytes	3	9.70 × 10^−2^

## 4. Discussion

CircRNAs are characterized by abundant expression, stable structure, and evolutionary conservation. In this study, circRNAs from CEFs mainly originated from chromosomes 1, 2, and 3, consistent with the study by Lei et al. on chicken muscle (22), Wang et al. on hen granulosa cells (23), and Zhang et al. on CEF and HD-11 cells (24). Our results showed that circRNAs from CEFs were mainly located in exons (80.8%), consistent with the findings of Wang et al. and Chen et al., who studied circRNAs from hen granulosa and DF1 cells (13, 23). Taken together, our results further enrich the map of circRNAs in chicken.

NDV is a major pathogen threatening the poultry industry. NDV infection was found to cause significant changes in the expression of many genes that are part of the immune response, which is important in clearing NDV infections. However, with the continuous development of scientific research, many unknown areas have emerged in the research on the pathogenesis of NDV, and the relationship between circRNAs and NDV infection is one of them. Prior to this study, Guo et al. used transcriptomics to explore the changes in circRNAs in the spleens of SPF chickens infected with the NDV-attenuated vaccine strain LaSota (25). They found that circRNAs were time-dependently expressed after vaccine inoculation in chicken thymic tissues. In the present study, we found that CEF infection with the velogenic NDV strain GM caused the differential expression of 86 circRNAs. We validated five of these DE circRNAs. The number of DE circRNAs in this study was higher than that reported by Guo et al. (*n* < 20), possibly because velogenic NDV strains induce a stronger immune response, resulting in more DE circRNAs.

The relationship between viral infections and circRNAs is complex. Li et al. demonstrated that the immune response and viral infections influence the biogenesis of host circRNAs (26). In addition, there is a competitive binding relationship between host circRNAs and viral mRNAs, suggesting that circRNAs have the potential to resist viral infection. Moreover, circRNAs may play a role in controlling various signaling pathways during viral infection. For example, the human circRNA_0050463 facilitates influenza A virus replication through sponging miR-33b-5p to regulate EEF1A1 (27). Wang et al. demonstrated that circ-chr19 plays the role of competitive endogenous RNA in Ebola virus infection by enhancing the expression of CLDN18 by targeting miR-30b-3p (28). In addition, Guo et al. predicted that multiple circRNAs are paired with gga-miR-6631-5p and that this miRNA can promote NDV replication in DF1, indicating that circRNAs may be involved in the regulation of NDV replication; however, this has not been verified (25). Intriguingly, our GO and KEGG enrichment results showed that circRNAs in the experimental group were enriched in metabolism-related pathways, while there were few innate immune-related pathways. In recent years, NDV has been found to affect the glucose (29), lipid (30), and amino acid metabolism of the host (31). Accordingly, our results suggest that circRNAs may be involved in host metabolic processes affected by NDV; follow-up studies should be conducted to confirm this.

Previous studies on the relationship between circRNAs and NDV infection mainly focused on the downstream miRNA and mRNA of circRNAs. In this study, we verified the effect of circ-EZH2 on NDV replication, demonstrating that circRNAs inhibit NDV infection in CEFs. EZH2 is the catalytic subunit of polycomb repressive complex 2 and plays a crucial role in many physiological or pathological activities, including the immune response (32). *EZH2* ablation has been reported to often compromise the effector CD8+ T cell response (33). Furthermore, Luo et al. demonstrated that EZH2 restricts *Tcf7* DNA methylation and promotes T_FH_ differentiation during acute viral infections (34). Further, Gao et al. revealed that ectopic expression of circ-EZH2 significantly promoted cell growth, migration, and invasion but inhibited cell apoptosis (35). Here, we predicted and identified three miRNAs that could bind to circ-EZH2 and found that those miRNAs had a total of 84 negatively correlated target mRNAs, including several metabolism-related genes. Among them, phosphatidylserine decarboxylase (PISD) is the target gene of the above-mentioned three miRNAs, which plays an important role in phospholipid substitution and organelle biogenesis (36). Glycerol kinase (GK), which is a key enzyme in lipid and carbohydrate metabolism and a multifunctional enzyme, showed predictive binding to two of these miRNAs (37). In addition, RhoH, CBLB, and TNIP1 are involved in apoptosis and inflammation (38–40), indicating that circ-EZH2 is involved in complex antiviral reactions during NDV infection. However, the specific mechanism underlying the effect of circ-EZH2 on NDV infection needs to be verified in future studies.

In conclusion, our study provides novel information regarding the transcription of circRNAs of CEFs upon velogenic NDV infection. Future studies should verify the functions of circRNAs during NDV infection.

## Data availability statement

The original contributions presented in the study are included in the article/Supplementary material, further inquiries can be directed to the corresponding authors.

## Author contributions

LC, BX, and TR designed the experiments. LC, JR, WD, JL, LF, and JC performed the experiments. LC, JR, and YC drafted the manuscript. CD, QL, BX, and TR reviewed and revised the manuscript. All authors have read and approved the final manuscript.
