# Supplementary material for: RNA sequencing reveals CircRNA expression profiles in chicken embryo fibroblasts infected with velogenic Newcastle disease virus
Source: Front Vet Sci. 2023 Mar 29;10:1167444. doi: 10.3389/fvets.2023.1167444 (PMC10090683; doi:10.3389/fvets.2023.1167444)
Supplement: Supplementary file 1 [file Table_1.DOCX]

Supplemental Table S1. The 86 differentially expressed circRNAs in the CEF-control group versus experimental group.

| CircRNA | log2(FC) | P-value | source_gene |
| --- | --- | --- | --- |
| novel_circ_000135 | -17.77436 | 0.03729663 | ncbi_395706 |
| novel_circ_000199 | -17.679406 | 0.04056403 | ncbi_418675 |
| novel_circ_000228 | 3.57244576 | 0.02036044 | ncbi_107057295 |
| novel_circ_000265 | -2.9679804 | 0.03886781 | NA |
| novel_circ_000281 | 3.46762597 | 0.02360799 | ncbi_418184 |
| novel_circ_000324 | 17.6876454 | 0.03674281 | ncbi_428366 |
| novel_circ_000412 | -2.1841768 | 0.00279179 | ncbi_107051666 |
| novel_circ_000635 | 2.40310649 | 0.01577214 | ncbi_420766 |
| novel_circ_000636 | 17.8936998 | 0.04468622 | ncbi_373953 |
| novel_circ_000711 | -18.274065 | 0.00755446 | ncbi_427256 |
| novel_circ_000843 | -2.3387324 | 0.0263099 | ncbi_107049475 |
| novel_circ_000869 | -3.7032565 | 0.0251066 | ncbi_396113 |
| novel_circ_000890 | -1.6368338 | 0.04748174 | ncbi_395593 |
| novel_circ_000922 | -17.679406 | 0.04054993 | ncbi_422038 |
| novel_circ_000929 | -2.8793777 | 0.01068815 | ncbi_101750635 |
| novel_circ_000968 | -1.6649459 | 0.03511879 | ncbi_418761 |
| novel_circ_001046 | -18.05263 | 0.01984998 | ncbi_423960 |
| novel_circ_001155 | 1.64829193 | 0.00284219 | ncbi_417102 |
| novel_circ_001184 | 18.0891926 | 0.01642125 | ncbi_422613 |
| novel_circ_001194 | -3.5008969 | 0.0438227 | ncbi_374052 |
| novel_circ_001232 | -17.982187 | 0.04577662 | ncbi_422033 |
| novel_circ_001411 | 3.3921833 | 0.00034595 | ncbi_395067 |
| novel_circ_001437 | 3.54360882 | 0.04679696 | ncbi_423988 |
| novel_circ_001438 | -3.6073389 | 0.03187329 | ncbi_420919 |
| novel_circ_001478 | -1.4367658 | 0.0294188 | ncbi_423548 |
| novel_circ_001579 | 17.9849954 | 0.04777214 | ncbi_421640 |
| novel_circ_001631 | 17.8890454 | 0.04594094 | ncbi_420919 |
| novel_circ_001674 | -17.816495 | 0.02141366 | ncbi_428458 |
| novel_circ_001741 | 3.25053764 | 0.0497322 | ncbi_422443 |
| novel_circ_001783 | 2.69991074 | 0.03057141 | ncbi_415722 |
| novel_circ_001830 | 2.047991 | 0.04165887 | ncbi_415834 |
| novel_circ_001888 | -18.270422 | 0.01183976 | ncbi_421461 |
| novel_circ_001932 | 3.71989193 | 0.01825605 | ncbi_396153 |
| novel_circ_002101 | 2.81073827 | 0.02035551 | ncbi_418184 |
| novel_circ_002136 | -1.1649919 | 0.03215242 | ncbi_420437 |
| novel_circ_002164 | -17.684889 | 0.03690675 | ncbi_419671 |
| novel_circ_002221 | -1.6977874 | 0.03944061 | ncbi_422949 |
| novel_circ_002291 | 18.0141633 | 0.01080856 | ncbi_418406 |
| novel_circ_002417 | -2.4923013 | 0.01555382 | ncbi_419453 |
| novel_circ_002473 | -18.425213 | 0.01180558 | ncbi_423662 |
| novel_circ_002558 | 1.05656358 | 0.04890141 | ncbi_423960 |
| novel_circ_002610 | -17.992586 | 0.04216016 | ncbi_420902 |
| novel_circ_002611 | 18.2332094 | 0.01275632 | ncbi_418406 |
| novel_circ_002623 | -2.419109 | 0.04480721 | ncbi_423814 |
| novel_circ_002629 | -18.247215 | 0.00554302 | ncbi_421134 |
| novel_circ_002647 | 18.650246 | 0.00349773 | ncbi_420194 |
| novel_circ_002830 | -2.212642 | 0.0015104 | ncbi_396303 |
| novel_circ_002911 | 1.25642269 | 0.0300301 | ncbi_424606 |
| novel_circ_003006 | 1.78131247 | 0.04303105 | ncbi_101751638 |
| novel_circ_003033 | -3.6523935 | 0.02221791 | ncbi_423194 |
| novel_circ_003152 | -2.8599886 | 0.02465739 | ncbi_421613 |
| novel_circ_003185 | -18.17042 | 0.01615347 | ncbi_427687 |
| novel_circ_003301 | 3.68235334 | 0.02742869 | ncbi_421798 |
| novel_circ_003420 | -18.146776 | 0.01007888 | ncbi_418449 |
| novel_circ_003546 | -2.8292842 | 0.02336025 | ncbi_427339 |
| novel_circ_003637 | 18.1313022 | 0.00816123 | ncbi_424158 |
| novel_circ_003700 | -1.5472359 | 0.01719564 | ncbi_415476 |
| novel_circ_003728 | 18.115371 | 0.02731269 | ncbi_421002 |
| novel_circ_003740 | 17.7103275 | 0.03154445 | ncbi_396205 |
| novel_circ_003850 | 17.5453344 | 0.04037416 | ncbi_107049603 |
| novel_circ_003945 | -2.3324518 | 0.04183686 | ncbi_100858912 |
| novel_circ_004077 | 3.10042002 | 0.04847571 | ncbi_420955 |
| novel_circ_004121 | 3.63830562 | 0.0375263 | ncbi_422095 |
| novel_circ_004164 | 1.35014354 | 0.01387309 | ncbi_420546 |
| novel_circ_004251 | 17.7103275 | 0.03154963 | ncbi_395067 |
| novel_circ_004256 | 1.2827953 | 0.04095932 | ncbi_417920 |
| novel_circ_004311 | 3.5258085 | 0.01814397 | ncbi_424004 |
| novel_circ_004341 | -1.129227 | 0.04488597 | ncbi_423048 |
| novel_circ_004370 | 1.61399959 | 0.02671728 | ncbi_423796 |
| novel_circ_004423 | 18.5054926 | 0.00716583 | ncbi_420686 |
| novel_circ_004432 | -2.146828 | 0.02012767 | ncbi_101750312 |
| novel_circ_004513 | 1.03730229 | 0.02547417 | ncbi_415612 |
| novel_circ_004550 | -17.992586 | 0.04208034 | ncbi_420580 |
| novel_circ_004700 | -1.0869524 | 0.04900832 | ncbi_417792 |
| novel_circ_004773 | 4.15661724 | 0.00361218 | ncbi_415515 |
| novel_circ_004780 | 2.09623896 | 0.04588998 | ncbi_418598 |
| novel_circ_004867 | 3.73714121 | 0.01634325 | ncbi_418832 |
| novel_circ_004930 | 3.25033696 | 0.00258045 | ncbi_395593 |
| novel_circ_004970 | 1.84880832 | 0.01298237 | ncbi_420663 |
| novel_circ_005212 | -1.942836 | 0.01257089 | ncbi_418781 |
| novel_circ_005222 | -2.1258835 | 0.02021653 | ncbi_418255 |
| novel_circ_005246 | 3.15123877 | 0.04909308 | ncbi_423194 |
| novel_circ_005300 | 17.9958161 | 0.03322478 | ncbi_424043 |
| novel_circ_005408 | -18.40874 | 0.04583524 | NA |
| novel_circ_005418 | -1.4704787 | 0.03497956 | ncbi_420784 |
| novel_circ_005651 | 2.61333457 | 0.02987715 | ncbi_417643 |
